# Building critical thinking in pre-service teachers: Dispositions, skills, and standards

**DOI:** 10.1371/journal.pone.0330536

**Published:** 2025-08-18

**Authors:** İpek Önal

**Affiliations:** Department of Educational Sciences, Faculty of Education, Akdeniz University, Antalya, Turkey; PNG National Research Institute, PAPUA NEW GUINEA

## Abstract

It is necessary for pre-service teachers to develop their critical thinking dispositions and skills in the pre-service period to develop similar skills in their future students. This research employed a one-group pretest-posttest design to investigate the effect of a 10-week critical thinking training on pre-service teachers’ critical thinking. The training had a significant effect on the development of critical thinking skills. While training led to a significant improvement in critical openness as a sub-dimension of critical thinking disposition, it did not affect critical skepticism. The results revealed significant differences in depth-breadth-adequacy and precision-accuracy dimensions of critical thinking standards.

## Introduction

Critical thinking (CT), used by a small part of societies throughout history, has become a skill that everyone must have [1]. Better problem-solving at the societal level, providing open and flexible thinking [2], and improving individuals’ economic, psychological, and vital well-being levels [3,4] show that CT is an essential skill for societies. The fact that individuals will need CT more in globalizing business environments [5] has gradually increased the importance of CT.

International sources recognize CT as a fundamental skill for the future workforce and quality education. Cruz and Dominguez [6] highlight that CT enhances the ability of recent graduates to communicate effectively, adapt to diverse conditions, solve problems, and make decisions in their professional lives. The World Economic Forum [5] identifies CT as one of the most in-demand cognitive skills for the future workforce in its Future of Jobs report, emphasizing the need for its prioritization in educational programs. The OECD’s [7] Future of Education and Skills 2030 project defines CT as essential for analyzing and effectively utilizing information, underscoring its necessity in educational processes. Similarly, UNESCO [8] advocates CT as a crucial competency that enables students to generate creative solutions to complex problems and actively take part in society. The 2015 Incheon Declaration further highlights the importance of quality education that fosters not only academic achievement but also CT skills, as emphasized by UNESCO and its educational stakeholders [9].

A qualified education approach for the future clearly emphasizes the necessity of CT. The increasing need for CT in modern life has made raising individuals as critical thinkers a much more crucial educational goal [10,11]. Today’s educational processes aim to develop individuals who skeptically approach new information and situations, produce ideas, conduct research, and critically evaluate [12].

Despite the emphasis on this skill, studies indicate that students struggle to develop it fully. Although individuals can develop CT early on, students have difficulty acquiring this skill at the primary school level [13]; students exhibit low performance and need guidance [14]. Similarly, Bouckaert [15] states that higher education students in OECD countries achieve low to moderate gains in CT skills. The underlying causes of this issue include inadequacies in current curricula [16], a lack of methodological knowledge for teaching CT [17,18], and insufficient teacher education [19–22].

In their systematic review, Bekcan et al. [23] emphasized that teachers’ inadequacy in implementing activities that support CT is one of the main difficulties in developing students’ CT skills. Despite recognizing the importance of CT, teachers often lack sufficient knowledge about which methods to integrate into their practices [24–26]. Similarly, Forawi [27] noted that teachers lack knowledge of teaching CT skills. As a priority for this problem, teachers need to acquire the knowledge, disposition, and skills necessary to foster CT in students [18,20,28–30]. Paul [31] emphasizes that teachers must be critical thinkers to teach CT effectively. However, studies indicate that in-service or pre-service teachers generally exhibit moderate or low levels of CT [32–35].

The meta-analysis study by Mamman et al. [36] reveals that pre-service teachers often possess low CT skills. Additionally, research indicates that pre-service teachers show not only low CT skills [37] but also limited dispositions toward CT [38]. Korkmaz [39] notes that teacher education programs do not sufficiently contribute to the development of CT in pre-service teachers.

In their systematic review, Taştı and Yıldırım [40] conclude that the CT skills of pre-service teachers are inadequate and that teacher education programs do not sufficiently focus on fostering CT dispositions. Lombardi et al. [13] highlights the importance of improving teachers’ access to training programs to enhance CT skills. Uslu [38], in a study examining the CT dispositions of pre-service teachers, underscores the need for more practices in teacher education programs that promote CT.

Low, Hu, and Cai [41] examine how pre-service teachers acquire CT skills, finding that they struggle to develop these skills and stressing the need for activities and effective strategies that encourage CT. Behar-Horenstein and Niu [17], in their review of 42 experimental studies on teaching CT, underline the necessity of structuring teacher education programs to support pre-service teachers’ CT skills continuously.

These findings highlight teacher education programs’ critical role in achieving the goal of cultivating critical thinkers in modern education [12]. To teach CT effectively, teachers must first become critical thinkers; thus, pre-service teacher education must prioritize developing these skills [42]. In this context, providing comprehensive training on CT skills within pre-service teacher education is a significant need, enabling future teachers to apply these skills in their classroom practices [17,19,43].

There is a need for practices to develop CT skills in teacher education programs. In this study, CT training was designed and implemented for pre-service teachers. The effectiveness of the training was evaluated in terms of CT skills, dispositions, and implementation of CT standards.

### Definition of critical thinking

Thinking, one of the basic functions of the mind, is a process in which ideas, concepts, and perceptions are organized based on the individual’s subjective consciousness [10]. According to Söylemez [44], thinking is a mental process in which the individual consciously and purposefully performs organized operations such as analysis, synthesis, evaluation, application, and conceptualization of the information they possess. In various definitions in the literature, thinking is described as a conscious exploration aimed at achieving a goal [45], as intentional cognitive behaviors performed to resolve events that disrupt an individual’s physical and mental balance [46], and as a rigorous examination to form ideas and draw conclusions [47]. Thinking is a component of four basic skills, categorized according to the mental operations involved [48]. These skills are creative thinking, problem-solving, decision-making, and CT.

CT, as a purposeful and controlled form of thinking, has its roots in Socratic questioning [49]. The term “critical” is derived from the Greek words *kriticos*, meaning “discerning judgment,” and *kriterion*, meaning “standard.” Consequently, CT is often defined as the ability to make decisions based on standards [47].

Originating in Ancient Greece, the concept of CT is now applied in various fields, including logic, ethics, health, psychology, and education. The modern foundations of CT can be traced back to John Dewey’s concept of “reflective thinking” [50]. Dewey described reflective thinking, the starting point for the modern definition of CT, as a conscious inquiry into the content, reasoning, and consequences of beliefs, ideas, or claims.

Due to its interdisciplinary and multifaceted nature, no single agreed-upon definition of CT exists. Norris [51] defines it as the process through which an individual evaluates their and others’ viewpoints based on appropriate standards. Ennis [52] defines CT as a skill that allows for the most accurate decision-making by examining the available evidence in the decision-making process. McKown [53] explains it as reflective skepticism, focusing on deciding what is reasonable or what action to take. McGregor [54] defines CT as a mental activity involving reviewing, identifying, and evaluating various forms of information, such as images, games, evidence, or opinions, to judge, infer conclusions, or derive meaning logically. Paul and Elder [4] describe CT as a systematic process to improve thinking by enhancing clarity, accuracy, consistency, and fairness. The common point of these definitions is that CT is a skill that enables individuals to approach and evaluate information in depth to reach logical judgments and decisions. In this context, CT ensures that information is understood through a critical lens, not superficially.

### Critical thinking skills and dispositions

The process of CT involves the application of specific skills and dispositions. Numerous sources discuss CT skills [11,55–59] and dispositions [60–67].

Within this scope, CT dispositions include open-mindedness, respect for others’ ideas, impartiality, independent thinking, seeking the truth, trusting reasoning, and intellectual humility. CT skills encompass interpreting, analyzing, making inferences, evaluating arguments, recognizing assumptions, carefully examining evidence, noticing fallacies, assessing the source’s reliability, distinguishing between claims and facts, and providing and requesting justifications.

Ennis [62] emphasizes that CT arises from the interaction of emotions and attitudes with cognitive skills and that individuals who lack a tendency for good thinking may fail to apply their skills actively. Similarly, Bailin [60] states that an individual who is inclined and has a high willingness to think critically may not be cognitively sufficient to demonstrate CT skills. Therefore, for an effective CT education, it is essential to simultaneously support both the cognitive and affective components of CT.

### Critical thinking in teacher education

CT is the ability to identify assumptions, present evidence-supported arguments, and draw logical conclusions based on available evidence. While scientists and educators agree on the importance of CT, there is a lack of consensus on how to teach it. In their systematic review, El Soufi and See [25] highlight that teaching CT at the undergraduate level in higher education remains limited. Barriers to learning CT at the undergraduate level include rote-based teaching, a lack of encouragement to question authority and evidence, and failure to teach CT clearly and structured. Paul [68] emphasizes that while students are expected to use intellectual standards, demonstrate appropriate dispositions, and analyze complex concepts, the necessary understanding and practices to support these abilities are lacking in higher education. There is a need to encourage the use of strategies and methods that will enable students to develop CT competence in higher education [69]. This need extends to teacher education programs. It is crucial for prospective teachers, who are expected to foster independent thinking in their future students, to recognize the extent to which they can think critically and understand their dispositions [32]. However, the inexperience of pre-service teachers in engaging with CT processes and the limited impact of courses that do not include practice make it difficult to learn and apply CT effectively [70]. Tok and Sevinç [71] state that applied, and experience-based learning methods are particularly effective in this process. Indeed, Öztürk [22] reveals in his study that activities and practices that will develop CT skills are given little or no place in the courses taken by prospective teachers during their undergraduate education. Studies evaluating the CT competencies of prospective teachers yield medium to low results. Koçer and Yangil [72] report that the CT dispositions of Turkish teacher candidates are at a medium level. Similarly, Durnacı and Ültay [73] found that the CT dispositions of teacher candidates are low.

These findings highlight the need for implementing more effective and comprehensive educational strategies in current teacher training programs to develop CT skills of pre-service teachers. The effective acquisition of CT skills will provide long-term benefits to teacher candidates, not only in academic terms but also in their professional development. In this way, pre-service teachers can continuously review and improve their future classroom practices, teaching methods, and student assessments [74]. More importantly, they will ensure that their future students do not merely memorize but also question, analyze, and develop their own ideas about what they have learned [22].

### Aim of the study

This study aims to determine the impact of CT training on the CT dispositions and CT skills of third-year English pre-service teachers enrolled in the education faculty of a public university. Accordingly, the following research questions are addressed:

1. What are the demographic characteristics of pre-service teachers?
2. How does CT training affect pre-service teachers’ CT dispositions scores from pre-test to post-test?
3. How does CT training affect the CT skills scores of pre-service teachers between pre-test and post-test?
4. How does CT training affect pre-service teachers’ CT standards scores from pre-test to post-test?

## Method

### Research design

This study is a single-group pretest-posttest quasi-experimental design. The measurements of the subjects concerning the dependent variables were obtained through a pretest before the experiment and a posttest after the experiment using the same subjects and measurement tools [75, p. 198]. This design was preferred to effectively evaluate the impact of the training developed as an experimental intervention. As stated by Creswell [76], the nature of the research often necessitates the use of a one-group experimental design in studies where new training is developed and implemented. Experimental research is a preferred scientific method in many fields because it allows for determining the effects of variables, clearly revealing cause-and-effect relationships, presenting results with concrete data, and making strong interpretations [77, p. 74]. While the CT training applied is an independent variable, CT dispositions, CT skills, and CT standards scale scores are the study’s dependent variables.

### Study group

The study group consisted of 16 pre-service teachers enrolled in the third year of the English Language Teaching program at the Faculty of Education of a public university in Turkey during the spring semester of the 2023–2024 academic year. Data on the demographic characteristics of the participants were collected for descriptive purposes and presented in the findings section. These data reveal the general profile of the sample group and the context of the study.

The experimental intervention was implemented in the “Critical and Analytical Thinking” course in the sixth semester of the English language teaching undergraduate program. Students who attended at least 10 weeks of the 14-week course and voluntarily participated were included in the study group.

### Data collection tools

The data for the study were collected using the “Critical Thinking Disposition Scale,” “Critical Thinking Skills Test,” and “Critical Thinking Standards Scale.”

#### Critical thinking disposition scale.

The Critical Thinking Disposition Scale was developed by Sosu in 2013 [78] and was adapted to Turkish by Akın and colleagues in 2015 [79]. The scale consists of eleven items and has two theoretical sub-dimensions: “critical openness” (items 1–7) and “reflective skepticism” (items 8–11). In Sosu’s [78] study, the Cronbach’s alpha coefficient for the entire scale was reported as .81. In the study by Akın and colleagues [79], the Cronbach’s alpha coefficient was found to be .78 for the entire scale,.68 for the critical openness sub-dimension, and .75 for the reflective skepticism sub-dimension. In the present study, Cronbach’s alpha value for the scale was .53 in the pre-test and .73 in the post-test. According to Morgan et al. [80, p. 131], alpha values below .70 are also acceptable for scales with a small number of items. Similarly, Akgül and Çevik [81] consider a Cronbach’s alpha value of .40 or above sufficient for the reliability of measurement tools.

#### Pamukkale critical thinking skills multiple choice form.

The Critical Thinking Skills Scale was developed by Duru et al. in 2022 [82] for university students. Unlike similar scales in the field, this scale evaluates CT skills through text analysis using ten different multiple-choice questions. The scale allows for an individual analysis of each CT skill. Overall Cronbach’s alpha internal reliability coefficient for the scale was reported as .92. In this study, the scale measured CT in terms of interpretation, analysis, evaluation, inference, self-regulation, and perspective-taking skills. The scale’s reliability for this study was found to be .41 for both the pre-test and post-test. Akgül and Çevik [81] regard a Cronbach’s alpha of .40 or above as acceptable for reliability. Cicchetti’s study [83, p. 286] on standardized test instruments in behavioral sciences stated that reliability coefficients between .40 and .59 indicate fair reliability in applied research settings.

#### Critical thinking standards scale.

The Critical Thinking Standards Scale was developed by Aybek et al. in 2015 [84] for pre-service teachers. The scale consists of forty-two items and has three theoretical sub-dimensions: “depth, breadth, and adequacy” (items 1–18), “accuracy and correctness” (items 19–30), and “importance, relevance, and clarity” (items 31–42). Cronbach’s alpha coefficient for the entire scale was reported as .75, while it was .89 for the first sub-dimension, .78 for the second, and.63 for the third. In the present study, the analysis revealed Cronbach’s alpha of .76 for the pre-test and .78 for the post-test [81].

### Data collection

Since the first weeks of the Critical and Analytical Thinking course covered topics such as course introduction, types of thinking, and analytical thinking, the implementation of CT training began in the fourth week. Students who volunteered to participate in the study were informed about the research before implementation. Tok and Sevinç [71] emphasized that CT skills require a long and detailed training process. The CT training lasted for 10 weeks (20 lesson hours). All three data collection tools were administered by the researcher before and after the implementation, with the application time of the tools lasting approximately 30–40 minutes.

#### The critical and analytical thinking course.

The Critical and Analytical Thinking course is an elective offered in the third year of the undergraduate program at the Faculty of Education. It is a theoretical course with two hours of instruction per week.

While designing the course, the general approach was adopted in teaching CT. This approach also called skill-based, focuses on CT knowledge, skills, and dispositions as its learning objectives [85, p. 1105]. The emphasis is placed on CT skills regardless of subject area [86]. The advantage of this approach is that students focus entirely on learning CT rather than spending their energy and time learning new information [87]. Existing research indicates that the general approach provides more substantial gains in teaching CT than other approaches [88,89]. Indeed, in their systematic review, El Soufi and See [25] considered the approach involving the teaching of general CT skills as one of the most promising methods.

The training aimed to improve pre-service teachers’ CT dispositions, skills, and application of standards. It was organized in 90-minute sessions for 10 weeks. The first week of the training was devoted to introducing the purpose and content of the training to the students and administering the pretests. In the last week of the training, post-tests were administered to measure the participants’ progress.

The course content includes: general thinking skills; analytical thinking; the concept of CT; tendencies of CT; intellectual virtues (intellectual humility, courage, integrity, etc.); affective traits related to bias and open-mindedness; fundamental concepts in CT (evidence, fact, assumption, premise, argument); CT skills; standards in CT (clarity, accuracy, precision, relevance, depth, breadth, significance, logic, fairness); elements of thinking (purpose, question, information, etc.); developing and evaluating arguments (recognizing the structure of arguments, analyzing in terms of purpose, question, assumptions, etc.); logical fallacies (evaluating case study texts within the framework of logical fallacies); asking questions that foster CT (lower- and higher-order questions, Socratic questioning techniques); methods and techniques (Six Thinking Hats, Socratic inquiry, Socratic circle, etc.); measuring and evaluating CT (standard tests and alternative methods); factors affecting CT; teacher behaviors and classroom environments that promote CT.

In the first four weeks of the training, the concepts of thinking and CT, CT dispositions, intellectual virtues and CT standards were addressed. In the next three weeks, CT skills, argument development, and logical fallacies were covered. The last weeks have focused on teaching practices that develop and evaluate CT. While structuring the course process, practical activities were planned each week within the framework of the topic, along with theoretical knowledge. For example, students prepared posters on intellectual virtues. They analyzed short texts regarding premises, assumptions, evidence, etc. Additionally, they evaluated arguments by recognizing the structure of the argument and developed their own arguments. They developed counterarguments by identifying logical fallacies in short texts about real-life problems. They participated in weekly class discussions guided by CT standards. They performed educational practices such as Socratic questioning and Socratic circle. At the end of the lessons, students were encouraged to question the changes in their thoughts and beliefs.

The researcher was the course instructor. Adequate training is crucial for educators conducting such interventions in CT education [25]. Since 2020, the researcher has been teaching courses on CT in teacher education programs. During the course implementation, a student-centered approach was adopted to design a learning process aligned with the nature of CT. Rather than simply delivering theoretical knowledge, practical activities were developed for each class, allowing students to actively engage in learning [90].

Studies highlighting the impact of classroom environments on CT skills and dispositions [63,87,91–93] were considered when planning the course process. For this reason, we discussed contradictory opinions and case studies/problems with alternative solutions during the class, and the instructor encouraged diverse and opposing perspectives. Students were motivated to articulate their views clearly and in detail and to evaluate opposing viewpoints critically. Throughout the course, student feedback on the conduct of learning activities was actively sought, and a democratic approach was adopted.

### Data analysis

The data were analyzed using SPSS (Statistical Package for Social Sciences) version 27.0. The normality of the data distribution was assessed using the Shapiro-Wilk test and skewness-kurtosis values, indicating that the pre-test and post-test scores for the CT disposition, skills, and standards scales followed a normal distribution. Based on this, the analysis employed descriptive statistics such as mean, frequency, and percentage, as well as the paired samples t-test. The significance level accepted as p < 0.05.

### Ethical considerations

Before the study, researcher obtained ethical approval (22.02.2024-861096) from the Scientific Research and Publication Ethics Committee of the university where the study would be conducted, along with institutional permission for its implementation. The data collection and program implementation process started on March 12, 2024, and ended on May 28, 2024. Additionally, students who agreed to participate in the study were informed about its purpose, and their written consent was obtained.

### Strengths of the study

It is essential to develop CT skills and dispositions simultaneously when teaching CT. Yu et al. [94] revealed in their study that there is a positive relationship between CT and dispositions. Therefore, educational processes should address both aspects concurrently to ensure students develop a comprehensive understanding of CT [95]. The researcher designed the educational process implemented in this study to foster CT skills and dispositions simultaneously. Rauscher and Badenhorst [96] noted that addressing these two components holistically is crucial to enable students to master both the cognitive and affective dimensions of CT. Similarly, the evaluation process was structured to assess students’ progress in both skill and disposition dimensions. Duru et al. [82] drew attention to the problem that CT skills and dispositions are often used synonymously in studies and are generally tested in evaluating programs that claim to support CT skills. In the present study, skills and dispositions were evaluated separately to assess the improvement in pre-service teachers’ CT by the end of the training. The scale developed by Duru et al. [82] allows for the analysis of participants’ application of these skills in terms of each skill of CT (interpretation, evaluation, etc.).

## Results

In the study, descriptive statistical analyses were initially conducted to examine the students’ demographic information. The differences between the pre-test and post-test scores of the CT dispositions, skills, and standards scales applied to the study group were examined. The findings from these analyses are presented below with corresponding tables and explanations.

In response to the first research question, the demographic characteristics of the pre-service teachers was examined. As shown in Table 1, the average age of the teacher candidates was 21.25 ± 1.12. Of the pre-service teachers, 62.5% were female (n = 11), and 38.9% were male (n = 7). All participants were graduates of Anatolian high schools (n = 16). Furthermore, 44% (n = 8) reported reading an average of 10–20 books per year.

**Table 1:** Descriptive findings on the demographic characteristics of the pre-service teachers.

Demographic information	N	%
Gender		
Female	10	62.5
Male	6	37.5
High School		
Anatolian High School	16	100
Number of Books Read Per Year		
0–10	5	31.2
10–20	6	37.5
20 and above	5	31.2
		Mean ± SD
Age	16	21.25 ± 1.12
Total	16	100

In response to the second research question, a paired samples t-test conducted on the pre-test and post-test mean scores of the critical thinking disposition scale revealed a significant difference in favor of the post-test (t = −2.423, p = .04 < 0.05). There was a significant improvement in the mean scores of the critical openness sub-dimension (t = 2.423, p = .02 < 0.05). Although there was an increase in the mean scores of the reflective skepticism sub-dimension after the training, this difference was not statistically significant (t = −1.243, p > 0.05). Overall critical thinking disposition (*d* = 0.55) and critical openness dimension (*d* = 0.60) achieved medium effect size [97], as shown in Table 2.

**Table 2:** Comparison of pre-test and post-test scores on the critical thinking disposition scale.

	Groups	N	Mean ± SD	df	t	p	Cohen’s *d*
Critical Thinking Disposition	Pre-test	16	42.00 ± 4.09	15	−2.423	.04	0.55
	Post-test	16	43.81 ± 4.91				
Critical Openness (subscale)	Pre-test	16	26.56 ± 2.94	15	−2.423	.02	0.60
	Post-test	16	27.68 ± 3.07				
Reflective Skepticism (subscale)	Pre-test	16	15.43 ± 1.63	15	−1.243	.23	
	Post-test	16	16.12 ± 2.50				

For the third research question, a paired samples t-test conducted on the pre-test and post-test mean scores of the pre-service teachers’ critical thinking skills scale revealed a significant difference in favor of the post-test (t = −3.989, p = .00 < 0.05), as shown in Table 3. Examining Cohen’s d value shows that critical thinking skills (*d* = 0.99) demonstrated a large effect size [97].

**Table 3:** Comparison of pre-test and post-test scores on the critical thinking skills scale.

	Groups	N	Mean ± SD	df	t	p	Cohen’s *d*
Critical Thinking Skills	Pre-test	16	25.31 ± 4.46	15	−3.989	.00	0.99
	Post-test	16	29.87 ± 3.59				

Within the scope of the fourth research question, a paired samples t-test conducted on the pre-test and post-test mean scores of the pre-service teachers’ critical thinking standards scale revealed a significant difference in favor of the post-test (t = −1.611, p = .00 < 0.05, d = 0.95), indicating a large effect size. Significant differences in favor of the post-test were also found in the depth-breadth-adequacy sub-dimension (t = −2.179, p = .04 < 0.05) and the precision-accuracy sub-dimension (t = −3.860, p = .00 < 0.05). No significant difference was observed in the importance-relevance-clarity sub-dimension (p > 0.05), as shown in Table 4. According to the calculated effect size values, after the training, the depth-breadth-adequacy dimension showed a medium effect (*d* = 0.53), and the precision-accuracy dimension showed a large effect (0.96) [97].

**Table 4:** Comparison of pre-test and post-test scores on the critical thinking standards scale.

	Groups	N	Mean ± SD	df	t	p	Cohen’s *d*
Critical Thinking Standards	Pre-test	16	161.87 ± 10.55	15	−3.804	.00	0.95
	Post-test	16	170.81 ± 11.36				
Depth-Breadth-Adequacy (subscale)	Pre-test	16	70.25 ± 6.09	15	−2.179	.04	0.53
	Post-test	16	73.37 ± 5.77				
Precision-Accuracy (subscale)	Pre-test	16	47.68 ± 5.09	15	−3.860	.00	0.96
	Post-test	16	51.87 ± 4.82				
Importance-Relevance-Clarity (subscale)	Pre-test	16	43.93 ± 3.33	15	−1.369	.19	
	Post-test	16	45.43 ± 3.99				

## Discussion

It is primarily necessary for teachers to be strong critical thinkers to raise students who are critically thinking in schools in the 21st century [98]. The fact that CT does not receive sufficient attention in pre-service teacher education [70] highlights the importance of evaluating the factors contributing to the CT of undergraduate teacher candidates. In this context, this study aimed to determine the effect of CT training on CT dispositions, skills, and the implementation of CT standards among third-year students at the faculty of education. Therefore, this study offers a more holistic evaluation by considering the effects of CT training on different components of CT together.

Regarding the second research question, which investigates CT dispositions, it was found that the pre-service teachers’ CT dispositions showed statistically significant improvements in the overall scale and the critical openness sub-dimension after the training. Although there was no significant difference in the reflective skepticism, an increase was observed at the end of the training. Educational interventions are essential in developing students’ knowledge, skills acquisition, and dispositions [99,100]. Various studies [101–104] have shown that targeted teaching activities positively affect developing pre-service teachers’ CT dispositions. In line with previous studies [25,89,105], the current research supports that explicit teaching of CT strengthens students’ CT dispositions in higher education.

Beyond the overall findings, it is also important to consider the results at the critical openness and reflective skepticism subscales. Critical openness is associated with being open to different ideas, opposing views, and perspectives, as well as understanding and seeking the truth. It is considered the most important predictor of CT disposition [106]. The results of this study indicate an increase in pre-service teachers’ critical openness following the training. This finding aligns with research suggesting that training pre-service teachers in CT contributes to their dispositions, particularly in seeking the truth and cultivating an open-minded approach [42,107,108]. However, this finding is not consistent with the results of Taghinezhad et al. [109], which reported no significant changes in certain dispositional aspects such as seeking the truth and open-mindedness following similar CT training. The reflective skepticism dimension as a CT disposition refers to an individual’s willingness to self-assess and regulate their beliefs, decisions, and actions [79]. The reflective skepticism improved following the training in the present study, but it was not statistically significant. As stated by Nuraini [110], pre-service teachers may possess CT dispositions, yet these have not been sufficiently developed, particularly regarding self-regulation. In contrast, previous research by Kurnaz and Sunbul [88] and Arı, Özsezer, and Tarım [111] suggest that CT instruction can positively contribute to students’ self-assessment within the CT process. The lack of significant development in reflective skepticism among student teachers in this study may indicate the need for longer-term application and reinforcement of certain CT dispositions. Supporting this notion, Demirbağ et al. [112] found that an 8-week CT training did not significantly alter pre-service teachers’ CT dispositions, suggesting that fostering such dispositions requires longer-term training. Although the training in this study was implemented for a long period such as 10 weeks, a longer period may be necessary to develop reflective skepticism in students. In fact, in their systematic review, Behar-Horenstein and Niu [17] stated that the longer the education period, the more observable the improvements in students’ CT became.

In response to the third question, which examines CT skills, the findings indicate that the training significantly improved the pre-service teachers’ CT skills. The parallel increase in CT dispositions and skills is another important finding indicating that the training was effective. The positive correlation between CT skills and CT dispositions [113] suggests that an increase in one tends to be accompanied by an increase in the other. For individuals to become critical thinkers, it depends on their acquisition of CT skills and willingness to use them, making them habitual [114]. Similar studies have emphasized that effective CT instruction involves the concurrent development of both dispositions and cognitive skills [115,116]. Palavan [42], Aybek [86] and Kong [117], in their experimental studies conducted with general approach to CT instruction at undergraduate level; revealed that CT dispositions and skills can be improved. In their systematic review, Tiruneh et al. [118] noted that experimental studies adopting a general approach improved both CT dispositions and skills. These findings are consistent with other experimental studies conducted with university students, revealing the positive effects of CT instruction through various interventions. Ekici [119] and Sutoyo, Agustini, and Fikriyati [120] found that online CT training was effective, while Alwehaibi [121] demonstrated that a CT program significantly enhanced university students’ CT dispositions and skills. Similarly, Kestel’s [122] meta-analysis revealed that programs designed to foster CT strongly improved students’ skills and dispositions. The findings of Lou’s study [123] on thinking education for university students align with the results of this study. While both skills and dispositions improved by the end of the training, the improvement in dispositions was more limited. In conclusion, an increase in both CT dispositions and skills was observed, suggesting that the training effectively developed both the cognitive and affective components of CT [42].

However, the low level of Cronbach’s alpha coefficient (.41) calculated for the Critical Thinking Skills Scale raises some concerns about its reliability. While this value is below the widely accepted reliability limits, it is important to consider the research context. When the items comply with homogeneity and consistency, the alpha can be relatively low [124, p. 235] depending on the number of items in the scale [125]. Indeed, Cicchetti [82] also classifies alpha coefficients between .40 and .59 as “moderately reliable” in applied research. Therefore, the alpha value obtained in our study should be interpreted considering both the number of items (10 items) and the sample size (16 people). Additionally, the study employed different measurement tools, and significant increases in these variables were observed. This multi-method approach allowed for a more holistic evaluation of the findings and reduced the risk of erroneous interpretation based on a single measurement tool.

Considering the fourth question, which investigates the implementation of CT standards, the results suggest an improvement in overall CT standards, depth-breadth-adequacy and precision-accuracy subdimensions. Likewise, Han and Brown [126] designed an intervention integrating the concepts, elements, standards, and characteristics of CT for pre-service preschool teachers. After the intervention, pre-service teachers improved their capacity to apply CT standards in their learning. Despite the improvements in other dimensions, no significant improvement was found in the importance-relevance-clarity dimension following the training.

## Limitations of the study

There were some limitations in the present study. The study data were limited to pre-service teachers from the Faculty of Education at a single university, which may limit the transferability of the results. Another limitation of the study is the relatively small sample size (n = 16), which can limit the generalizability of the findings. Also, the findings should be considered based on the study’s one-group design, which lacks a comparison group. The study focused solely on teaching CT through the general approach. The duration of the CT training was limited to 10 weeks. Future experimental studies could be designed with a control group and extended over a longer period to examine the effects of different approaches to teaching CT. Finally, as the data in this study were gathered solely through quantitative tools, future research could explore the development of students’ CT skills and dispositions using qualitative or mixed methods.

## Future directions and recommendations

This study presents the experimental results of CT training as a separate course in teacher education, using a general approach. The findings provide evidence for the positive effects of CT training, targeting the dimensions of skills, dispositions, and standards simultaneously. It is suggested that effective CT training should focus not only on cognitive skills but also on developing dispositions. Furthermore, this study may serve as a guide for educators responsible for designing courses incorporating CT in pre-service teacher education and organizing professional development activities for teachers. It offers insights into effective approaches and a better understanding of the elements for integrating CT into education.

It is recommended that follow-up studies be conducted at different time intervals to examine the long-term retention of CT skills and dispositions. The long-term effects of the training can be explored through mixed-methods research. Qualitative research methods such as surveys, interviews, observations, and focus group studies can be used to understand participants’ experiences with CT.

Throughout the training, this study implemented active learning strategies such as opinion development, collaborations, debates, and problem-solving activities. Future CT training delivered through digital materials and interactive platforms may positively influence students’ CT skills and dispositions. Additionally, the impact of CT training provided using various teaching strategies and methods aligned with the nature of CT can be examined.

In future training sessions, emphasizing students’ self-assessment and self-regulation processes may help develop reflective skepticism disposition. Methods should be integrated to encourage students to question their thinking processes. Research indicates that 8 weeks and longer training programs effectively develop CT dispositions. Ensuring the continuity of the training by practitioners will contribute to a better understanding of the long-term effects of training on dispositions. Another recommendation is that CT training be planned for a more extended period and supported by practical activities. Finally, students’ lack of improvement in the dimension of importance-relevance-clarity shows that they have difficulty implementing these standards. In future education programs, concrete examples and practical exercises can be provided for students to use these standards more effectively.
